# Use of Plastics with Hot Food among Saudi Pregnant Women Is Associated with Increased Concentrations of A1C, Thyroid-Stimulating Hormone, and Homocysteine and Decreased Concentrations of Vitamins and Minerals

**DOI:** 10.3390/nu12092609

**Published:** 2020-08-27

**Authors:** Mudi H. Alharbi, Walaa A. Mumena, Sahar A. Hammouda

**Affiliations:** Clinical Nutrition Department, College of Applied Medical Sciences, Taibah University, P.O. Box 344, Madinah 42353, Saudi Arabia; mhjharbi@taibahu.edu.sa (M.H.A.); saharhammouda@hotmail.com (S.A.H.)

**Keywords:** plastics, hot food, TSH, HCY, A1C, micronutrients

## Abstract

Data regarding association between the use of plastics with hot food and levels of vitamins and minerals, and other biochemical parameters are lacking. Cross-sectional data for 740 healthy pregnant Saudi women were collected from 21 health care centres and 2 hospitals from Madinah, Saudi Arabia. Detailed data regarding the frequency of plastic use with hot food were collected, and laboratory analyses of thyroid-stimulating hormone (TSH), homocysteine (HCY), glycated A1C (A1C), and selected vitamins and minerals were also done. Daily use of plastics with hot food was frequently reported among young mothers (*p* = 0.002). Plastic use with hot food on a daily basis was positively associated with TSH, HCY, and A1C, while it was negatively associated with concentrations of vitamin E, zinc, and selenium. Future research should address the complex hormonal and metabolic abnormalities that are linked to the release of certain components associated with the use of plastics with hot food. Interventions are urgently needed to eliminate the use of plastics with hot food to prevent health complications that may result from the long-term use of these materials.

## 1. Introduction

Food contamination is one of the most serious global concerns [1]. Accordingly, any contamination of food matter, even prior to birth, can be a serious hindrance to health and/or the preservation of life. The causal factors of this contamination can be living and non-living agents. These agents, labelled food hazards, exist in foods and cause many health impacts for those who consume them [2]. Typically, the contamination may happen due to living microbes or non-living chemical toxification [1]. In the Gulf countries, the statistics in relation to food contamination are a cause for concern. According to the Gulf Petrochemicals and Chemicals Association, Saudi Arabia was responsible for 67% of the Gulf Cooperation Council (GCC) production of plastic resins, and it was also the largest consumer of polymers, accounting for 61% of the 5 million tons consumed in the GCC, with the United Arab of Emirates being the next-largest consumer at 19% [3]. It is known that people in Saudi Arabia use 20 times more plastic (especially plastic bags) than the global average [4]. According to the Saudi Standards, Metrology and Quality Organization (SASO) in 2017, 16 types of polypropylene and polyethylene products under 250 microns thick had been banned. However, food-manufacturing plastics have been exempted from the ban, with the exception of grocery bags or bags used to handle hot food items [3,4].

Plastic has become an integral part of our everyday lives as it adds ease and comfort to our lives [5]. However, its dark side has revealed it to be the most common persistent organic environmental pollutant on earth. Plastic, thus, has become one of the biggest health threats to humanity as toxic chemicals leach out of plastics and enter our food chain [6]. This has created a big question mark over the safety and quality of food materials ready for consumption. Since the use of plastics is ubiquitous, its use as cutlery and utensils as well as food packaging has become common practice [7]. It has been proven that merely eating and/or drinking using plastic utensils/containers is a severe health risk, and more so when food in plastic containers is heated in microwaves [1,8]. Harkin [9] has called it a ‘vector for contamination’ and suggested that there is a bioaccumulation of plastics inside our bodies. Plastic has been reported to release harmful chemicals into foodstuffs whenever it is used [10,11]. Examples of these contaminants include polyethylene, plasticizers from polyvinyl chloride (PVC), acetaldehyde from polyethylene terephthalate (PET), polychlorinated biphenyls, bisphenols A, S, and F, phthalates (diethyl-phthalate—DEHP) from polymers, acrylonitriles, dioxins, parabens [10], and styrene from polystyrene [5,11]. Of these, phthalates and bisphenols are the two materials most commonly used in the manufacture of food containers and tableware, which is a hazardous practice [12]. All these chemical exposures or additives are known to cause serious diseases among humans. For example, they act as carcinogens and promote metastasis [5]. They also cause heavy metal toxicity [7,13], suppression of the immune system [14], common birth defects [10], fertility-related disorders [9], obesity, allergies, cardio-, hepato- and cumulative nephrotoxicity, and neurodegenerative disorders [5,15]. They are also known to cause developmental problems in children [16]. Additionally, pregnant women are particularly susceptible to the effects of these contaminants, which may eventually affect the fetus [10]. Pregnant women who are exposed to phthalates may be at high risk of preterm birth [17].

This effect could even start before conception, as elucidated by a recent study by Zhang et al. [18]. Bisphenol and phthalates have been suspected to be endocrine disruptors and are known to impact Body Mass Index (BMI), particularly during pregnancy [19]. Since they are able to cross the placenta and reach the cord blood and amniotic fluid, it can therefore be assumed that they may have long-term and short-term outcomes in offspring as well. The long-term complications include type 2 diabetes and cardiovascular disorders, among many others. Unfortunately, most of us are completely unaware of the potential for harm caused by the regular use of plastics [9]. Identifying the problem in plastics can facilitate the production of better-quality plastics, which cause less or no leaching of contaminants and minimal loss of nutrients when used as containers for food items heated in microwaves. However, information on the influence on nutrients of heating foods in plastic containers is extremely limited and it needs to be proven, even though there are many studies that suggest problematic effects of microwavable plastics, microwave heating, packaging methods, and storage temperatures on food [10]. However, studies that measure whether nutrient levels are hampered by exposure to plastic are lacking. Thus, this study aimed to assess the relationship between the use of plastics with hot food and the levels of specific micronutrients and other biochemical parameters among healthy first-trimester pregnant Saudi women.

## 2. Materials and Methods

This cross-sectional study was extracted from a larger prospective study that aimed to detect preventable risk factors that may cause birth defects. The study was conducted between May 2009 and January 2011 among 1034 healthy first-trimester pregnant Saudi women (in the first 13 weeks of gestation as determined from last menstrual period and confirmed by pregnancy test or ultrasound) who visited one of the participating clinics (21 health care centres and 2 hospitals) in Madinah, Saudi Arabia. Mothers with poor pregnancy outcomes (having major or minor congenital malformations, intrauterine fetal deaths or neonatal deaths for un-identified reasons) were excluded from this study, as congenital malformation has been reported to be associated with abnormal levels of some biochemical parameters [20]. Demographic, biochemical, and anthropometric data were collected from all participants. Data regarding the frequency of use of plastics with hot food were also collected. The main question items addressed the use of plastic dishes when heating food and purchases of hot food in plastic bags or plastic containers.

### 2.1. Biochemical Parameters

Participants were instructed to arrive early after an overnight fast, and venous blood samples were withdrawn and placed into EDTA tubes. The preparation of samples included centrifugation for plasma separation into 8 dark Eppendorf tubes and freezing at −80 °C until the time of analysis.

Blood A1C levels were measured using standard procedures by the NycoCard A1C, which is a boronate affinity assay. A thyroid-function test, including only thyroid-stimulating hormone (TSH), was measured by a competitive immunoassay commercial kit, which was manufactured in Spain by BioKit S.A. (Barcelona, Spain). Reference intervals were determined as TSH, 0.27–4.2 mIU/L. The inter- and intra-assay coefficients of variation were ≤9.1% and ≤5.6%. Total homocysteine (HCY), folic acid, and vitamin B_12_ concentration in the plasma were measured by sandwich ELISA (enzyme-linked immunosorbent assay) using commercial kits according to the instructions provided by the manufacturer (IBL, Internatonal, Hamburg, Germany). Concentrations of plasma retinol (vitamin A) and α-tocopherol (vitamin E) were determined using reverse-phase high performance liquid chromatography (HPLC) using a UV detector at 292 nm for vitamin E and 325 nm for vitamin A. Selenium was measured by spectrophotometer (atomic absorption) on M5 SOLAAR (Thermo Electron, Cambridge, UK) using a GF95 graphite furnace with auto-sampler using Argon as a purging gas. The zinc assay was measured by a zinc bioassay kit that was designed to directly measure zinc in biological samples with no requirement for pretreatment. This method utilized a chromogen that forms a colored complex with zinc. The intensity of the color was measured at 425 nm and was directly proportional to zinc levels in the sample. Magnesium was measured by a magnesium bioassay kit that was designed to directly measure magnesium in biological samples with no requirement for pretreatment. A calmagite dye in the kit forms a colored complex with magnesium. The intensity of the color was measured at 500 nm and was directly proportional to magnesium levels in the sample.

Participants were instructed to arrive early after an overnight fast, and venous blood samples were withdrawn and placed into EDTA tubes. Preparation of samples included centrifugation for plasma separation into 8 dark Eppendorf tubes and freezing at −80 °C until the time of analysis.

Blood glycated A1C (A1C) levels were measured using standard procedures by the NycoCard A1C, which is a boronate affinity assay. A thyroid-function test, including only TSH, was measured by a competitive immunoassay commercial kit, which was manufactured in Spain by BioKit, S.A. Reference intervals were determined as TSH, 0.27–4.2 mIU/L. The inter- and intra-assay coefficients of variation were ≤9.1% and ≤5.6%. Total homocysteine (HCY), folic acid, and vitamin B12 concentration in the plasma were measured by sandwich ELISA (enzyme-linked immunosorbent assay) using commercial kits according to the instructions provided by the manufacturer (IBL, Internatonal, Hamburg, Germany). Concentrations of plasma retinol (vitamin A) and α-tocopherol (vitamin E) were determined using reverse-phase HPLC at 292 nm for vitamin E and 325 nm for vitamin A. Selenium was measured by spectrophotometer (atomic absorption) on M5 SOLAAR (Thermo Electron, Cambridge, UK) using a GF95 graphite furnace with auto-sampler using Argon as a purging gas. The zinc assay was measured by a zinc bioassay kit that was designed to directly measure zinc in biological samples with requirement for pretreatment. This method utilized a chromogen that forms a colored complex with zinc. The intensity of the color was measured at 425 nm and was directly proportional to zinc levels in the sample. Magnesium was measured by a magnesium bioassay kit that was designed to directly measure magnesium in biological samples with no requirement for pretreatment. A calmagite dye in the kit forms a colored complex with magnesium. The intensity of the color was measured at 500 nm and was directly proportional to magnesium levels in the sample.

Consent forms were signed by all participants during their first visit to the clinic. Ethical approval to conduct this study was obtained from the Ethics Committee at King Abdulaziz City for Science and Technology (project number: AT.28-113).

### 2.2. Statistical Analysis

Descriptive data are presented as mean ± standard deviation (SD) for continuous variables, while data for categorical variables are presented as frequency (%). Chi-square testing was used to assess the association between two categorical variables, and the Kruskal–Wallis H test was used to compare the dependent variables across the different groups (based on plastic use). Frequency of the use of plastics was coded as follows: never use (0), use monthly (1), use weekly (2), and use daily (3). Multiple linear regression analysis of plastic use with hot food (independent variable) on biochemical parameters was included in this study adjusting for age. A significance level of <0.05 with two-tailed testing was adopted in this study. Statistical analysis was done using SPSS 20 (SPSS Inc., Chicago, IL, USA).

## 3. Results

**Figure 1:**
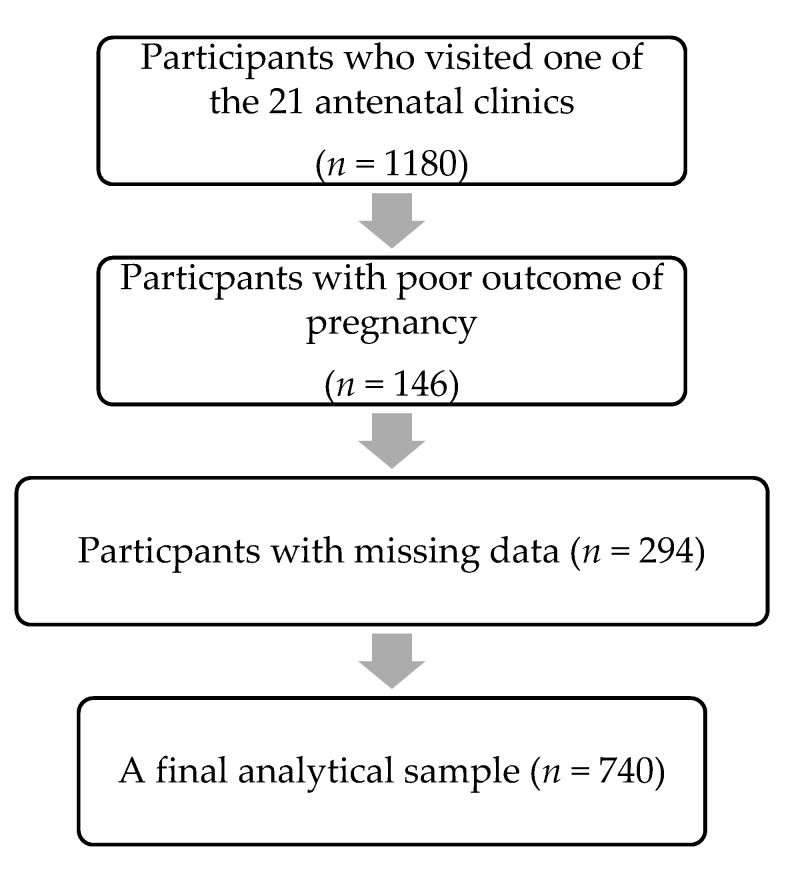
Flowchart of participant selection.

A total of 740 pregnant women were included in the final analysis of this study after excluding mothers with missing data (see Figure 1). The mean age of these pregnant women was 27.8 ± 6.16 years, and 29.2% of them lived with smoking husbands (*n* = 240). 11% (*n* = 78) of mothers reported daily use of plastics with hot food, 39.1% (*n* = 289) and 19.6% (*n* = 145) reported weekly and monthly use of plastics with hot food, respectively, while 30.8% (*n* = 228) reported no use of plastics with hot food. General characteristics of first-trimester pregnant Saudi women according to the frequency of use of plastics with hot food are presented in Table 1. All the characteristics included in this study were similar across the different groups, except that pregnant women who reported using plastics with hot food daily were significantly younger (*p* = 0.002).

The means of the selected biochemical parameters (TSH, HCY, and A1C concentrations) of first-trimester pregnant Saudi women based on the frequency of use of plastics with hot food were significantly higher among daily users of plastics with hot food, while zinc and selenium concentrations were significantly lower among daily users compared to the other groups (non-users, weekly users, monthly users) (see Table 2).

The data obtained from the multiple linear regression analysis show that plastic use with hot food on a daily basis was positively associated with TSH (*p* = 0.004), HCY (*p* = 0.001), and A1C (*p* = 0.005), while it was negatively associated with concentrations of vitamin E (*p* = 0.027), zinc (*p* = 0.010), and selenium (*p* = 0.005) (see Table 3).

## 4. Discussion

Daily use of plastics with hot food was frequently reported among young mothers. Plastic use with hot food on a daily basis was positively associated with TSH, HCY, and A1C, and negatively associated with concentrations of vitamin E, zinc, and selenium.

The invention of plastics has made our life simpler and more comfortable, due to its multiple uses, making it ubiquitous across many human activities [5]. This suggests that almost all of the population is expected to use plastic plates for feeding for the sake of convenience [21]. This was also true in our case to some extent: almost 70% of our study population were using plastic plates for reheating purposes or buying hot food in plastic containers. Similar observations of Australian pregnant mothers were reported by Callan et al. [22]: 65% of them were heating food in plastic containers. However, lower frequencies were observed by Mariscal-Arcas et al. [23] and Valvi et al. [24] in their studies of expectant mothers from Spain, only 52% of whom were using plastic containers for heating food. Of these, the majority used plastics rarely—at a frequency of less than once a month in both studies, which differed from our results, where the majority of women were using plastics weekly. This lower frequency was similar to the usage pattern of 657 pregnant Spanish women, as reported by Casas et al. [25]. Regarding the 30% of our studied sample population who were not using plastic for reheating food at all, it is assumed that they have a high awareness of the issues posed by reheating food on microwavable plastic plates and that this is the main reason for their non-use. However, there is no confirmation of this as there was no significant difference in the levels of educational qualification across the groups. Furthermore, according to SASO, most of the food and/or packaging commonly used in Saudi Arabia is made from PET and polypropylene (PP), which are approved by the US Food and Drug Administration (FDA) as food contact substances (FCS) [26]. However, other types of low price plastics are still available in the market and could be the type used with hot foods in some food service facilities, especially in low-income communities, where less enforcement of food-related law is applied. Moreover, Polystyrene (PS) is still used widely in some restaurants, food facilities, and food shops, as well as Polycarbonate (PC) and phthalates, which could explain the results of a study conducted in Saudi Arabia that found high levels of urinary phthalate metabolites among children. In addition, this high level of urinary phthalate was positively associated with the frequent consumption of foods that had been exposed to phthalates [27]. This result of the latter study demonstrates the truth or existence of using this type of plastic with food in some extent in Saudi Arabia.

Pacyga et al. [12] considered maternal age and BMI as factors that correlate with differing plastic usage patterns, and this formed the basis for the inclusion of these characteristics in order to understand the dynamics of our study population. Our results show that, besides age, no tested maternal characteristics, such as the educational qualifications of the pregnant women and their pre-pregnancy BMI, correlated with the variation in frequency of plastic usage. However, none of the existing reports studied an association of these maternal characteristics with the frequency of use of plastic plates. From our results, it can be concluded that age has a critical role in awareness on plastic usage: with age, people become more health-conscious and highly resistant to using plastic.

The analysis of our results displays a mix of significant and insignificant variation in the levels of the biochemical parameters tested within the groups segregated according to the frequency of use. In the case of serum TSH, HCY, and A1C, an increase was observed in the levels with respect to increase in plastic usage frequency, while serum zinc and selenium showed a statistically significant reverse trend: their levels fell with greater use of plastic plates for heating food. Even though the levels of TSH and HCY are within the normal range, this increase with plastic usage indicates that this is not a healthy practice as higher levels of both of these are known to increase the risk of recurrent miscarriages [28,29]. In addition, Pacyga et al. [12] have reported that bisphenol A (BPA) and phthalates present in plastics impact TSH levels in pregnant women. However, Aung et al. [30] observed that TSH and BPA were inversely associated with each other, contradictory to our results. It can be seen that plastic-induced endocrine disruptions that affect the body’s hormones either mimic or block hormones such as estrogens by interacting with estrogen receptors [31]; thyroid hormones are affected by inhibiting the uptake of the thyroid hormones by transporter monocarboxylate transporter 8 in the brain or [32] by changing thyroid histology or altering its weight [33,34], and pancreatic hormones are affected by increasing pancreatic beta-cell insulin content in an estrogen-receptor-dependent manner [35,36]. In general, by interfering with hormone actions, they may affect reproductive development and metabolism, affect insulin use in the body, and have other possible impacts.

Minerals such as zinc and selenium have been reported to be essential for multiple human body functions [2]. Coming to the effect of plastics on zinc, it has been reported by Rowdhal and Chen [15] that phthalates, which is a type of plastic, showed a lower levels of serum zinc, as was also observed in our case: the serum zinc levels were lower in the group using that used plastic plates daily. The lower level of nutrients may be an indirect effect of increased A1C, TSH, and HCY. Hammouda and Mumena [37] and Kutbi et al. [38] reported a positive correlation of A1C with TSH and HCY and a negative relation with vitamin B_12_, folate, and vitamin E, in the first-trimester-pregnant Saudi women, observations similar to ours. Along with this, a negative relation was also observed between A1C and minerals such as zinc and selenium in expectant mothers in their first trimester by Hammouda et al. [39], also similar to our results. However, there was no information on their plastic usage frequency. It can also be concluded that this increase in TSH and HCY, along with the decrease in vitamin E, and minerals such as zinc and selenium, could be due to the increased level of A1C. However, it is still not advisable to use plastic plates for heating purposes. Thus, it becomes extremely important that people become at least aware of the negative impact of the use of heated plastics and follow the safety guidelines regarding their usage.

To the best of our knowledge, this is the first study to investigate the association between the use of plastics with hot food and micronutrients and biochemical parameters. However, this study is limited by the unavailability of data concerning the classification of the plastics (e.g., safe vs. not safe) used by the study participants and of the analysis of the specific chemicals emitted when heating plastics.

## 5. Conclusions

Hormonal and metabolic abnormalities were found to be associated with the use of plastics with hot food, and this should be addressed in future research. Interventions are urgently needed to eliminate the use of plastics with hot food to prevent health complications that may result from the long-term use of these materials.

## Figures and Tables

**Table 1:** General characteristics of Saudi pregnant women by frequency of using plastics with hot foods (*n* = 740).

	Daily Use (*n* = 78)	Weekly Use (*n* = 289)	Monthly Use (*n* = 145)	Never Use (*n* = 228)	*p*
Age, years, mean ± SD					
Pregnant women	26.6 ± 5.66	27.0 ± 5.94	29.0 ± 6.00	28.2 ± 6.52	0.002 ^1^
Education level of pregnant women, *n* (%)					
≤Primary education	16 (9.82)	61 (37.4)	29 (17.8)	57 (35.0)	0.321
<University degree	40 (11.0)	141 (38.6)	65 (17.8)	119 (32.6)	
≥University degree	22 (10.4)	86 (40.6)	51 (24.1)	53 (25.0)	
Smoking, spouse, *n* (%)	30 (12.5)	93 (38.8)	44 (18.3)	73 (30.4)	0.654
Pre-pregnancy BMI, kg/m^2^, mean ± SD	25.1 ± 4.80	24.8 ± 5.53	25.9 ± 5.88	25.3 ± 5.43	0.284

^1^ Significance level of <0.05.

**Table 2:** Mean values of selected biochemical parameters of Saudi pregnant women based on the frequency of using plastics with hot foods (*n* = 740).

	Daily Use (*n* = 78)	Weekly Use (*n* = 289)	Monthly Use (*n* = 145)	Never Use (*n* = 228)	*p*
TSH, mIU/L	2.01 ± 0.62	1.82 ± 0.58	1.82 ± 0.54	1.75 ± 0.57	0.019 ^1^
HCY, μmol/L	7.57 ± 1.01	7.22 ± 0.93	7.21 ± 0.84	7.10 ± 0.88	0.005 ^1^
A1C, %	5.07 ± 0.33	4.97 ± 0.32	4.98 ± 0.31	4.93 ± 0.32	0.020 ^1^
Magnesium, mg/dL	4.26 ± 0.67	4.34 ± 0.67	4.31 ± 0.66	4.38 ± 0.64	0.455
Vitamin B_12_, pmol/L	539 ± 62.4	550 ± 64.5	553 ± 49.7	556 ± 56.2	0.084
Folate, nmol/L	53.3 ± 7.77	54.4 ± 7.71	55.0 ± 6.18	55.3 ± 7.08	0.139
Vitamin E, mg/L	29.9 ± 3.91	30.7 ± 3.91	30.8 ± 3.65	31.1 ± 3.80	0.097
Vitamin A, µg/L	676 ± 103	702 ± 101	701 ± 97.4	709 ± 100	0.102
Zinc, μg/dL	101 ± 13.3	104 ± 14.3	103 ± 13.2	106 ± 14.7	0.020 ^1^
Selenium, ng/mL	11.4 ± 2.56	12.0 ± 2.54	12.0 ± 2.29	12.4 ± 2.50	0.019 ^1^

^1^ Significance level of <0.05. TSH, thyroid-stimulating hormone; HCY, homocysteine; A1C, glycated A1C.

**Table 3:** Multiple linear regression analysis of plastic use with hot foods on selected biochemical parameters among Saudi pregnant women (*n* = 740).

	B	SE	*p*	95% Confidence Interval	*R*-Square
TSH, mIU/L	0.06	0.02	0.004 ^1^	0.02 to 0.10	0.01
HCY, μmol/L	0.11	0.03	0.001 ^1^	0.04 to 0.17	0.02
A1C, %	0.03	0.01	0.005 ^1^	0.01 to 0.06	0.01
Magnesium, mg/dL	−0.03	0.02	0.216	−0.08 to 0.02	<0.01
Vitamin B_12_, pmol/L	−4.22	2.15	0.051	−8.44 to 0.01	0.01
Folate, nmol/L	−0.52	0.26	0.051	−1.04 to 0.00	0.01
Vitamin E, mg/L	−0.31	0.14	0.027 ^1^	−0.58 to −0.03	0.01
Vitamin A,µg/L	−6.89	3.65	0.060	−14.1 to 0.28	0.01
Zinc, μg/dL	−1.33	0.51	0.010 ^1^	−2.34 to −0.32	0.01
Selenium, ng/mL	−0.26	0.09	0.005 ^1^	−0.04 to −0.02	0.01

^1^ Significance level of <0.05; all models were adjusted for age.
